# Identification and Characterization of a Small-Molecule Inhibitor of Death-Associated Protein Kinase 1

**DOI:** 10.1002/cbic.201402512

**Published:** 2014-11-07

**Authors:** Theis S Wilbek, Tine Skovgaard, Fiona J Sorrell, Stefan Knapp, Jens Berthelsen, Kristian Strømgaard

**Affiliations:** a Center for Biopharmaceuticals, Department of Drug Design and Pharmacology, University of Copenhagen, Universitetsparken 2 2100 Copenhagen (Denmark); b Novo Nordisk A/S, Novo Nordisk Park 2760 Måløv (Denmark); c Nuffield Department of Clinical Medicine, Structural Genomics Consortium, University of Oxford, Old Road Campus Research Building Roosevelt Drive, Oxford OX3 7DQ (UK); d Costerton Biofilm Center, Institute for International Health, Immunology and Microbiology, Faculty of Health Sciences, University of Copenhagen (Denmark)

**Keywords:** drug discovery, enzymes, inhibitors, phosphorylation, protein structures

Death-associated protein kinase 1 (DAPK1) has been associated with *N*-methyl-d-aspartate (NMDA) receptor-mediated excitotoxicity, and has recently been suggested as a target for the treatment of stroke. Here, we examined DAPK1-mediated in vitro phosphorylation of the GluN2B subunit of NMDA receptors. We established a high-throughput screening assay for the protein kinase DAPK1 by using a Caliper microfluidics capillary electrophoresis system (Caliper Life Sciences/PerkinElmer) and identified a novel small-molecule imidazo-pyramidazine inhibitor (**6**) targeting the catalytic domain of DAPK1. The inhibitor was characterized by enzyme kinetic assays and isothermal titration calorimetry (ITC), and a high resolution crystal structure provided detailed insight into the binding mode of the inhibitor.

The human genome comprises more than 500 different kinases, the majority of which have been linked to regions associated with various diseases by chromosomal mapping.^1^ Thus, clearly kinases have a vast potential as drug targets, but so far only relatively few kinase inhibitors have been approved for non-oncology applications. This is often ascribed to the notorious challenge of developing selective kinase inhibitors. DAPK1 has been associated with cell death mediated by apoptosis^2^ and has been suggested as a novel target for treatment of stroke because of its involvement in NMDA receptor-mediated excitotoxicity, which is believed to be the major cause of brain damage following a stroke.^3^ In addition, DAPK1 has recently been suggested as a drug target in stroke therapy because of its interaction with p53.^4^ Specifically, it has been suggested that DAPK1 increases the flux of Ca^2+^ ions through NMDA receptors composed of GluN1 and GluN2B subunits by DAPK1-dependent phosphorylation of Ser1303 in the C-terminal region of GluN2B.^3^ A cell-penetrating peptide fragment of GluN2B (GluN2B_1292–1304_, KKNRNKLRQHSY) reduced stroke in an animal model, an effect that was attributed to inhibition of the DAPK1/GluN2B interaction.^3^ Attempts have also been made to indentify small-molecule inhibitors of DAPK1,^5^ and two X-ray crystal structures of small-molecule inhibitors binding to DAPK1 are avaliable (see the Supporting Information).5a–b

It is well-established that excitotoxicity is primarily mediated by NMDA receptors containing GluN2B; NMDA receptors containing GluN2A subunits do not play a prominent role in this process. This important difference between the two subunits, GluN2A and GluN2B, has been shown to be related to differences in their intercellular C-terminal domains (CTDs).^6^ In general, differences in the CTD of GluN2A and GluN2B are responsible for a range of complex neuronal and behavioral manifestations and are therefore of importance for understanding subtype-specific roles of NMDA receptors.^7^

Several kinases have been suggested to phosphorylate the CTDs of GluN2A and GluN2B and thereby regulate NMDA receptor function. Specifically, Ca^2+^/calmodulin-dependent protein kinase II (CaMKII) has been suggested to phosphorylate Ser1303 of GluN2B,^8^ a residue that is also targeted by protein kinase C (PKC).^9^ CaMKII and DAPK1 share a number of notable structural and functional properties. In particular both kinases harbor a calmodulin-binding sequence and an autoregulatory domain (ARD) next to the catalytic domain and are therefore regulated by calmodulin (or proteins with calmodulin-like domains).^10^ There are also notable differences between the two kinases, for example DAPK1 is active only in its non-phosphorylated form,^11^ whereas activation of CaMKII is more complex.^12^ Both kinases have been suggested to bind directly to the CTD of GluN2B near Ser1303 and thus co-localize with GluN2B.^3^, ^13^ Thus it is proposed that phosphorylation of Ser1303 and co-localization are responsible for the regulation of calcium influx by NMDA receptors (Figure 1). To investigate the mechanistic details of DAPK1/NMDA receptor interactions further, including differences between GluN2A and GluN2B, we used in vitro phosphorylation assays. First the catalytic domain of DAPK1 (residues 1–285) was cloned,^14^ expressed in *Escherichia coli*, and purified; the reference substrate Peptide382d (KKRPQRRY**S**NVF) and the 13-mer peptide N2B_13_ (**1,** GluN2B_1292–1304_, KKNRNKLRQH**S**Y; Figure 2 A) were prepared. The in vitro phosphorylation assay (see the Supporting Information) showed that, as expected, peptide38 was an excellent substrate for DAPK1, whereas surprisingly phosphorylation of **1** was strongly repressed (Figure 2 B). Thus, clearly Ser1303 in **1** is not phosphorylated by DAPK1 in vitro.

**Figure 1:**
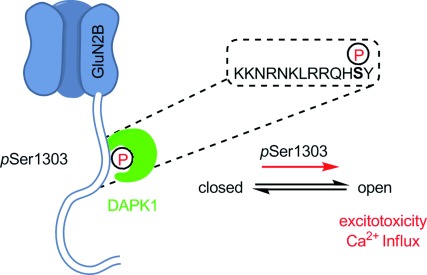
Proposed mechanism for GluN2Bs increased excitotoxicity after phosphorylation by DAPK1. After phosphorylation of Ser1303 DAPK1 remains bound to the phosphorylation site with its catalytic domain, thereby mediating increased calcium influx.

**Figure 2:**
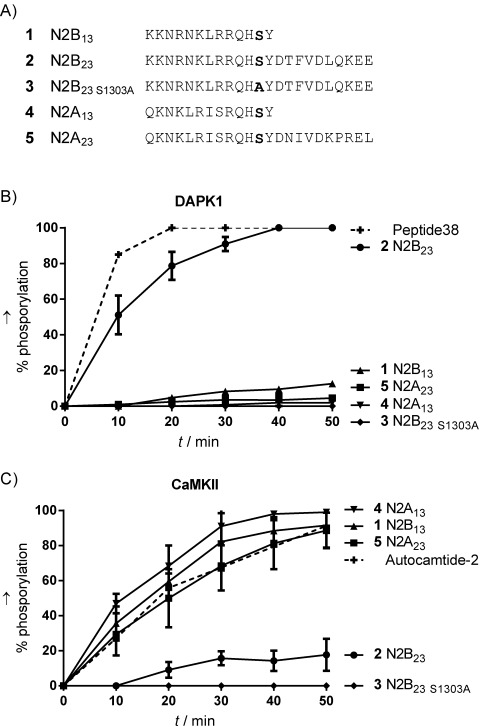
Comparison of peptide substrates containing Ser1303 (GluN2B) or Ser1291 (GluN2A). A) Alignment of peptides used in in vitro phosphorylation studies. B) Phosphorylation by DAPK1. C) Phosphorylation by CaMKII. Control substrates are shown with dashed lines. Degrees of phosphorylation were assessed by analytical HPLC (*n*=3, mean±SD). Concentration: peptide 50 μm, ATP 100 μm, kinase concentration was adjusted to fit the timescale. For further details see the Supporting Information.

This prompted us to examine related peptides derived from GluN2B and GluN2A (Figure 2 A) and we compared in vitro phosphorylation of these peptides by using both recombinant DAPK1 and CaMKII. Interestingly, for the 23-mer peptide N2B_23_ (**2,** GluN2B_1292–1314_; KKNRKKNRNKLRRQH**S**YDTFVDLQKEE), which has ten additional C-terminal amino acids, we observed phosphorylation to a degree similar to that for the reference substrate. Substituting Ser1303 with Ala (**3**, N2B_23 S1303A_, KKNRKKNRNKLRRQH**A**YDTFVDLQKEE) prevented phosphorylation, thus suggesting that Ser1303 was indeed phosphorylated. We examined the inhibitory effect of **1**, a putative inhibitor of Ser1303 phosphorylation, on peptide **2**, but did not observe any effect (data not shown). Next we studied the corresponding 13- and 23-mer GluN2A peptides, N2A_13_ (**4**, GluN2A_1280–1292_, QKNKLRISRQH**S**Y) and N2A_23_ (**5**, GluN2A_1280–1302_, QKNKLRISRQH**S**YDNIVDKPREL); for both, Ser1291 corresponds to Ser1303 in GluN2B. Interestingly, none of these peptides was phosphorylated by DAPK1, thus indicating that DAPK1 indeed selectively phosphorylates Ser1303 in the GluN2B CTD. We also examined these peptides for in vitro CaMKII phosphorylation; we observed an almost reversed substrate specificity compared to DAPK1: GluN2A derived peptides **4** and **5** were phosphorylated at a similar rate and to the same degree as the control peptide, Autocamtide-212a (KKALRRQE**T**VDAL-NH_2_), the shorter N2B_13_ (**1**) was phosphorylated in a similar manner. In contrast, neither the longer GluN2B peptide, N2B_23_ (**2**), nor its Ala mutant, **3**, was phosphorylated by CaMKII. Together, these results indicate that DAPK1 might be responsible for the phosphorylation of Ser1303 in GluN2B, thus validating DAPK1 as a target for inhibition of selective GluN2B CTD phosphorylation; CaMKII phosphorylates the corresponding residue (Ser1291) in GluN2A. Furthermore, it was seen that substrate recognition by DAPK1 is dependent on additional amino acids C-terminal to the phosphorylation site compared to CaMKII substrates, and that these additional residues seemingly inhibit recognition by CaMKII.

Next, we identified small-molecule compounds that inhibit DAPK1 and are thus potential leads for the development of compounds with neuroprotective properties and potential application for the treatment of stroke. For this, we established a high-throughput enzymatic screening assay for DAPK1. First we prepared a fluorescent peptide substrate, with 5-carboxyfluorescein (5FAM) coupled to the N-terminus of peptide38 (5FAM-Peptide38; see the Supporting Information). This peptide was used in a Caliper microfluidics capillary electrophoresis assay, which separates phosphorylated peptide product from the non-phosphorylated peptides in microcapillaries. The fluorescently labeled peptide enabled quantification in real time and thus determination of reaction velocities. To ensure that the kinase reaction was at steady-state and that initial velocities were measured, the DAPK1 concentration was adjusted to achieve a substrate turnover of no more than 15 % throughout the time course of the reaction. The *K*_m_ for ATP was determined (1.24 μm, Supporting Information), and screening was carried out with an ATP concentration of approximately 2×*K*_m_, to allow ATP-competitive DAPK1 inhibitors to be discovered. The quality of the screening was determined, and the resulting *Z*-factor of 0.84 was deemed satisfactory. We then screened approximately 4300 compounds from a kinase-targeted in house compound collection in duplicate at 10 μm. The compound library contained compounds presumed to have an increased probability of binding to the conserved ATP-binding pocket of protein kinases. The compound collection included 244 known, potent kinase inhibitors from the InhibitorSelect library (Millipore). From the first screening, we identified 96 compounds exhibiting more than 58 % inhibition of DAPK1; these were selected for determination of IC_50_ values with the same assay. Of the 96 compounds, 57 were known kinase inhibitors from the CAL library; the remaining 39 were potentially novel inhibitors of DAPK1. The 96 compounds showed IC_50_ values ranging from 0.003 μm (staurosporine) to 131 μm. Of these, 26 had IC_50_ values below 1 μm: 22 were from the InhibitorSelect library (Supporting Information), and the most potent of the others was compound **6** (IC_50_=0.247 μm).

**Figure:**
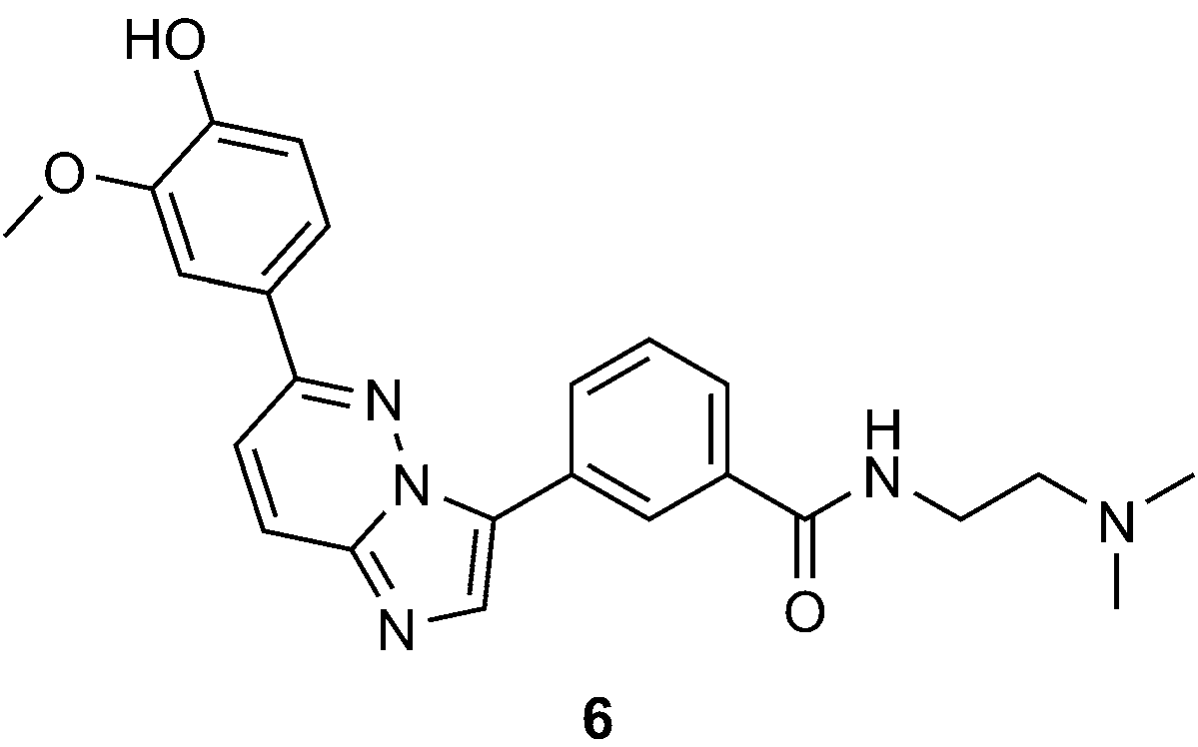


Compound **6** was tested in primary screening against a number of other kinases (SRC, BLK, AKT3, JAK2, PAK4, PAK7, PIM1, PIM2, CAMKI, and CAMKII); 60 % inhibition at 10 μm compound was used as the cut-off limit. PIM1, JAK2, PAK7, and SRC were identified, and IC_50_ values from the subsequent assays were determined as 1.6, 1.8, 5.6, and 0.15 μm, respectively. Notably, only SRC was inhibited to the same extent as DAPK1; CAMKII did not to pass the primary screening. Thus **6** was clearly an interesting compound. Therefore, we verified the inhibitory activity of **6** towards DAPK1 in a different assay: the equilibrium dissociation constant (*K*_d_) of **6** was determined by ITC to be 0.24±0.09 μm (Figure 3), thus verifying that **6** indeed binds DAPK1 at nanomolar concentrations. Additionally, **6** showed inhibition in the in vitro phosphorylation assay (Supporting Information).

**Figure 3:**
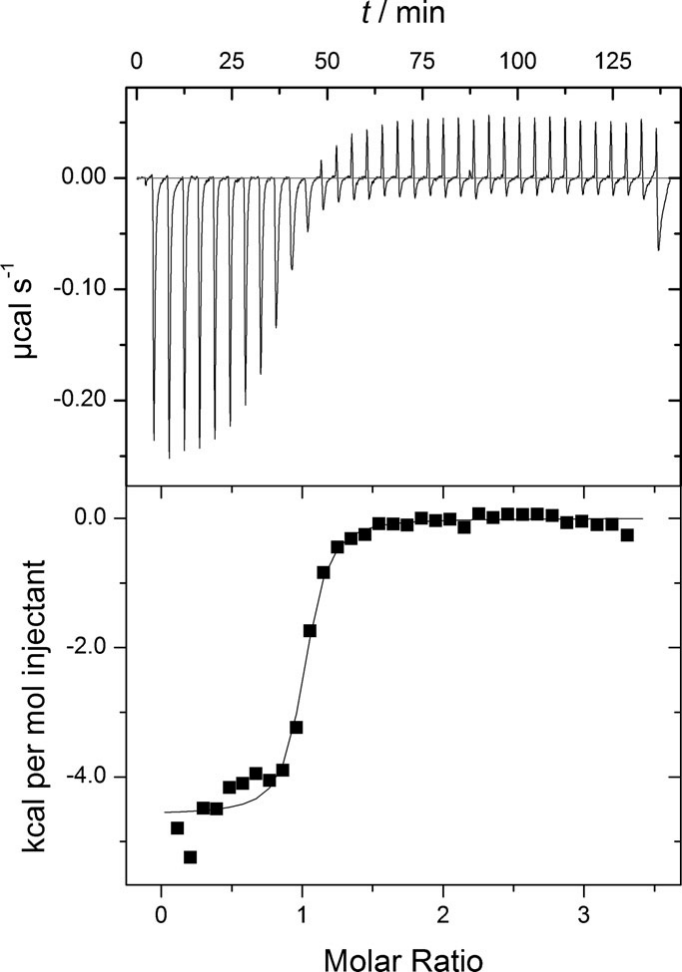
Representative ITC of the catalytic domain of DAPK1 (residues 1–285) into 6. ITC reveals an equilibrium dissociation constant (*K*_d_) of 0.24± 0.09 μm (*n*=4).

Finally, we were interested in the binding mode of **6** to DAPK1, and we therefore co-crystallized DAPK1 with **6**. Our structure knowledge for small-molecule inhibitors of DAPK1 is currently limited to two published X-ray crystal structures (aside from the broad kinase inhibitor staurosporine):5a, b One of the inhibitors is a ruthenium-based metal complex;5a the other X-ray structure contains only part of the small-molecule inhibitor.5b Thus, clearly there is interest in examining the molecular details of small-molecule inhibition of DAPK1. Gratifyingly, we obtained an X-ray co-crystal structure at 1.9 Å in space group *P*2_1_2_1_2_1_ with one molecule in the asymmetric unit (PDB ID: 4TXC; Figure 4 and Supporting Information). All residues were clearly visible in the electron density map, with the exception of the C-terminal residues Lys278 to Ser285, which were disordered in the structure. After initial molecular replacement, electron density corresponding to a bound ligand was immediately visible at the ATP binding pocket, and following several rounds of refinement the structure of **6** was modelled into the electron density with high confidence (Figure 4 B). The electron density for the dimethylamino-ethylamide moiety of **6** was weak in relation to the aromatic part, most likely due to increased flexibility of this part of the molecule in general, and specifically this moiety extended outwards from the binding pocket and did not interact with DAPK1. In contrast, the heterocyclic part of **6** was relatively planar and slotted into the ATP binding pocket in a manner typical for type I ATP-competitive kinase inhibitors: it formed a key hydrogen bond between the backbone amide of Val96 in the kinase hinge region and N-7 of the imidazo-pyramidazine portion of the ligand (Figure 4 C). The hydroxylmethoxyphenyl group of **6** occupied back pocket I^15^ of the ATP-binding site and was held in position by several polar interactions, including hydrogen bonds with the side chain of Lys42 in the kinase N lobe and Glu64 of helix C (Figure 4 C). In the absence of an inhibitor, the salt-bridge formed between these two residues typically stabilizes the DAPK1 active conformation.^16^ The ligand hydroxyl moiety was also within hydrogen bonding distance of the backbone amide of Phe162 (part of the kinase DFG motif). In DAPK1 and closely related kinases (DAPKs, MLCKs, TRIOs), this residue is locked in an “in” conformation, thus leading to constitutive assembly of the regulatory (“R′′) spine.^16^ In general there was good shape complementarity between the ligand and the active site (Figure 4 D); however, visual inspection of the adjoining areas indicated that it might be possible to modify the ligand to improve both specificity and efficacy. For DAPK1, several important substrate-recognition motifs have been identified: GEL, PEN and PEF/Y motifs (Figure 4 A).^17^ Although the aliphatic arm of **6** was close to the PEN and GEL motifs, no direct contact was made. It is plausible that extension of the ligand towards one of these motifs could improve its potency by interfering with the substrate recognition site. An alternative approach would be extension into the pocket next to the ATP site, as demonstrated by several non-competitive type III kinase inhibitors.^18^

**Figure 4:**
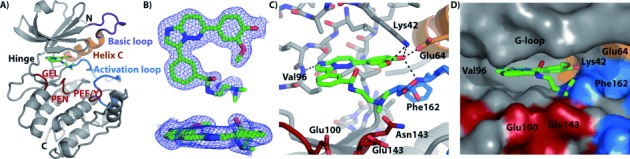
DAPK1–CPR005231 (6) co-crystal structure (PDB ID: 4TXC) A) Structural model and ligand binding site showing the basic loop (purple), helix C (orange), activation loop (blue), substrate binding motif residues (red), and ligand (green). B) Electron density map contoured at 1 *σ* for the ligand shown from two perspectives. (2*F*_o_−*F*_c_) C) Detail of the ligand-binding site showing key interactions and proximity to DAPK1 substrate-binding motif residues. D) Surface-filled representation of the ligand-binding site illustrating nearby pockets for potential ligand modification.

In conclusion two different approaches to develop an inhibitor of DAPK1 were explored: a peptide-based approach based on mimicking the ARD and a more classic small molecule approach. The in vivo effect of the cell penetrating version of GluN2B_1292–1304_ (N2B13, **1**) was explained by mimicking the ARD and binding directly to the catalytic domain of DAPK1;^3^ however no in vitro inhibitory effect was seen for **1**, thus suggesting that the effect could be mediated by interfering with other interactions.^19^ The small-molecule screening approach led to the identification of **6**. This displayed some selectivity towards DAPK1 and reasonable potency, and from the X-ray co-crystal structure we identified the molecular details of the interaction of **6** with DAPK1. Thus, **6** exhibits a range of promising properties for further studies, and the structure suggests specific areas for modification for improving both selectivity and potency.

## Supporting Information

As a service to our authors and readers, this journal provides supporting information supplied by the authors. Such materials are peer reviewed and may be re-organized for online delivery, but are not copy-edited or typeset. Technical support issues arising from supporting information (other than missing files) should be addressed to the authors.

miscellaneous_information
